# Field-based measurement of cardiorespiratory fitness to evaluate physical activity interventions

**DOI:** 10.2471/BLT.18.213728

**Published:** 2018-09-12

**Authors:** Justin J Lang, Emily Wolfe Phillips, Heather M Orpana, Mark S Tremblay, Robert Ross, Francisco B Ortega, Diego Augusto Santos Silva, Grant R Tomkinson

**Affiliations:** a Centre for Surveillance and Applied Research, Public Health Agency of Canada, 785 Carling Ave, Ottawa, K1S 5H4 Canada.; b Healthy Active Living and Obesity (HALO) Research Group, Children’s Hospital of Eastern Ontario Research Institute, Ottawa, Canada.; c School of Kinesiology and Health Studies, Queen’s University, Ontario, Canada.; d Promoting Fitness and Health through physical activity research group (PROFITH), Department of Physical Education and Sports, Faculty of Sport Sciences, University of Granada, Granada, Spain.; e Research Center in Kinanthropometry and Human Performance, Federal University of Santa Catarina, Florianopolis, Brazil.; f Kinesiology and Public Health Education, University of North Dakota, Grand Forks, United States of America.

The World Health Organization (WHO) recently developed a *Global action plan on physical activity* aimed at attaining a 15% relative reduction in the global prevalence of insufficient physical activity in adolescents and adults by 2030.^1^ The plan involves a multifaceted approach to create a society that intrinsically values and prioritizes policy investments in physical activity as a regular part of everyday life.^1^ An important component of this plan is the evaluation process for monitoring global changes in the prevalence of insufficient physical activity and for assessing the global plan’s impact and related efforts. For surveillance, WHO has previously used self-reported physical activity measures, such as the Global Physical Activity Questionnaire.^2^ Although self-reported physical activity has several benefits related to feasibility and obtaining an understanding of the context in which individuals are active, self-reporting could be susceptible to response biases, hindering its validity.^3^ Some researchers and surveillance systems are moving towards more objective measures, such as pedometers or accelerometers. Under ideal conditions, objective measures can provide more accurate estimates of overall physical activity levels, although they are not always feasible and pose challenges related to increased cost and time required for data collection and analysis.^4^ As an alternative, cardiorespiratory fitness is a physical trait that is primarily determined by physical activity behaviours. Here we provide our perspective on using cardiorespiratory fitness as an objective measure that could complement the evaluation process of the global plan by providing a proximal outcome of physical activity levels in individuals of all ages and for countries across income categories.

## What is cardiorespiratory fitness?

Cardiorespiratory fitness represents an intermediate variable between physical activity behaviours and health outcomes that reflects the capacity of numerous bodily organs, such as the heart, lungs and muscles, to support energy production during physical activity and exercise.^5^ Although 30–50% of cardiorespiratory fitness is determined by genetics, habitual physical activity remains the primary means of improving fitness; which is therefore a proximal outcome of physical activity levels.^5^ From a public health perspective, cardiorespiratory fitness provides a robust measure, because of its low month-to-month variability within individuals. Cardiorespiratory fitness may provide a stable reflection of recent physical activity levels, similar to glycosylated haemoglobin, reflecting glucose control over a period of several months. 

The gold standard measure of cardiorespiratory fitness remains laboratory-based assessments with gas analysis. Cardiorespiratory fitness is often reported as maximal oxygen uptake (*V*O_2max_) in adults, peak oxygen uptake (*V*O_2peak_) in children and adolescents or is standardized as metabolic equivalents.^5^^,^^6^ However, laboratory-based testing is costly and impractical for population-based assessments with large samples. Among adults, several field-based measures have been used to estimate cardiorespiratory fitness. For instance, the time-to-complete 800 to 1500 m runs are popular in Asia, whereas the 12-minute distance walk and/or run and the 2 km walk tests are popular in Europe and North America. For at-risk populations, the 6-minute walk test (that is, distance covered in 6 minutes) is commonly used.^6^ Among children and adolescents, the 20 m shuttle run test is a widely used field-based assessment, with a recent study reporting results from 50 countries, including 16 low-income countries.^7^ Furthermore, the 20 m shuttle run test has been proposed as a suitable and feasible health indicator for international surveillance in children and adolescents.^5^ These types of field-based tests have been incorporated into surveillance systems in some high- and middle-income countries, although further evidence describing implementation and feasibility in countries across income categories is needed. Due to of the low cost of equipment, no need for specialized human resources, the ability to assess multiple participants simultaneously and easily interpretable results, field-based cardiorespiratory fitness assessments are promising. Although agreement is needed on optimal field-based tests across the lifespan, cardiorespiratory fitness tests provide suitable objective measures that could complement existing evaluation processes for physical activity interventions, programmes and strategies.

## Cardiorespiratory fitness and health

A large body of evidence supports cardiorespiratory fitness as a strong and independent predictor of cardiovascular disease and all-cause mortality.^6^ In adults, cardiorespiratory fitness has been monitored in some settings, because of the improved prediction of cardiovascular disease when cardiorespiratory fitness is added to traditional risk scores, such as the Framingham risk score.^6^ Furthermore, a meta-analysis found that a 1-metabolic equivalent increase (corresponding to a sustained 1 km/h increase in walking or jogging speed) in cardiorespiratory fitness, was associated with a 13% and 15% reduction in cardiovascular disease and all-cause mortality risk, respectively.^8^ The majority of these cardiorespiratory fitness related benefits are seen when individuals move from the least fit category to the next least fit category, suggesting that the majority of the benefits from cardiorespiratory fitness are gained when physically inactive individuals become physically active.

Among children and adolescents, cardiorespiratory fitness is associated with health, independent of self-reported physical activity levels, indicating that cardiorespiratory fitness provides additional insight into the health of children and adolescents beyond the predictive power of self-reported physical activity levels alone.^5^ Studies have consistently identified strong associations between cardiorespiratory fitness and cardiometabolic profiles, where fitter children and adolescents often have better glucose tolerance, cholesterol and triglyceride levels and lower blood pressure.^9^ More importantly, cardiorespiratory fitness in childhood tracks moderately well into adulthood, providing possible insight into the future health of a population. For instance, a systematic review concluded there was strong evidence that cardiorespiratory fitness levels in childhood predict cardiovascular risk profiles, including metabolic syndrome and arterial stiffness, in adulthood.^10^ Supporting these conclusions, a large cohort study of approximately 700 000 participants with a median follow-up period of 34 years identified a graded association between high cardiorespiratory fitness levels in late adolescents and a decreased risk of myocardial infarction in adulthood.^11^

## Improving cardiorespiratory fitness

In physically inactive adults, who likely also have low baseline cardiorespiratory fitness, 30 minutes of sustained brisk walking (< 50% of maximum heart rate) 3–4 times per week, over a minimum period of 12 weeks, can result in clinically meaningful improvements in cardiorespiratory fitness (5–15% increase).^6^ These improvements are often seen following a standardized physical activity programme, regardless of age, sex, ethnicity and baseline cardiorespiratory fitness in previously inactive individuals.^6^ Generally, higher intensities, longer durations, and/or more frequent bouts of physical activity result in larger improvements in cardiorespiratory fitness. Individuals with high baseline cardiorespiratory fitness often need higher intensity exercise (> 85% of maximum heart rate) to improve cardiorespiratory fitness levels. Among children and adolescents, a 12-week exercise programme, on average, results in an 8–9% increase in cardiorespiratory fitness, independent of sex, age and maturation.^12^ Thus, a 12-week programme structured around the WHO physical activity guidelines^13^ would likely induce a meaningful change in cardiorespiratory fitness levels, regardless of age, sex and ethnicity.

## Implementation

The WHO STEPwise approach to noncommunicable disease surveillance provides a conceptual framework to help countries select core risk factors for health surveillance, and to use standard methods to collect, analyse and disseminate the surveillance information.^14^ The adult risk factor framework describes three steps, with step 1 being the easiest to implement and step 3 being the most demanding. Step 1 involves questionnaires (demographics, self-reported physical activity); step 2 provides direction for physical measurements (body mass, height, blood pressure); and step 3 includes biochemical measurements (fasting blood glucose, total cholesterol). Step 2 of the STEPwise approach provides an optional component for the assessment of physical fitness, representing an opportunity to expand and build the foundation for cardiorespiratory fitness surveillance through selecting standardized assessments and developing the resources needed to guide countries in their surveillance efforts. Building this foundation and encouraging the collection of baseline data could help facilitate the measurement of cardiorespiratory fitness as an objective assessment to complement the global plan’s evaluation process. Further discussions and studies are required to assess the feasibility of implementing field-based tests of cardiorespiratory fitness in countries across income categories, including considerations of cultural acceptability, the physical environment and the costs and barriers to implementation. Careful consideration is needed regarding the safety and health of those surveyed, including safety during testing, as well as ethical considerations of follow-up with individuals identified as at-risk.

Ultimately, the goal of decreasing physical inactivity is to improve the health of all populations and cardiorespiratory fitness is an important mediating variable between physical activity and health. Feasibility issues remain in incorporating objective measures of physical activity, and there are known challenges with self-reported physical activity measures. The objective measurement of cardiorespiratory fitness as a proximal outcome of total physical activity levels could be used to complement the global plan’s evaluation process. While more research is needed, it is an opportune time to further explore the measurement of cardiorespiratory fitness in an international context. 
